# Supplementary material for: AI-based antibody discovery platform identifies novel, diverse, and pharmacologically active therapeutic antibodies against multiple SARS-CoV-2 strains
Source: Antib Ther. 2024 Sep 26;7(4):307–23. doi: 10.1093/abt/tbae025 (PMC11456866; doi:10.1093/abt/tbae025)
Supplement: Moldovan_Loomis_et_al_2024-Supplemental_Material_tbae025 [file moldovan_loomis_et_al_2024-supplemental_material_tbae025.docx]

**SUPPLEMENTAL**


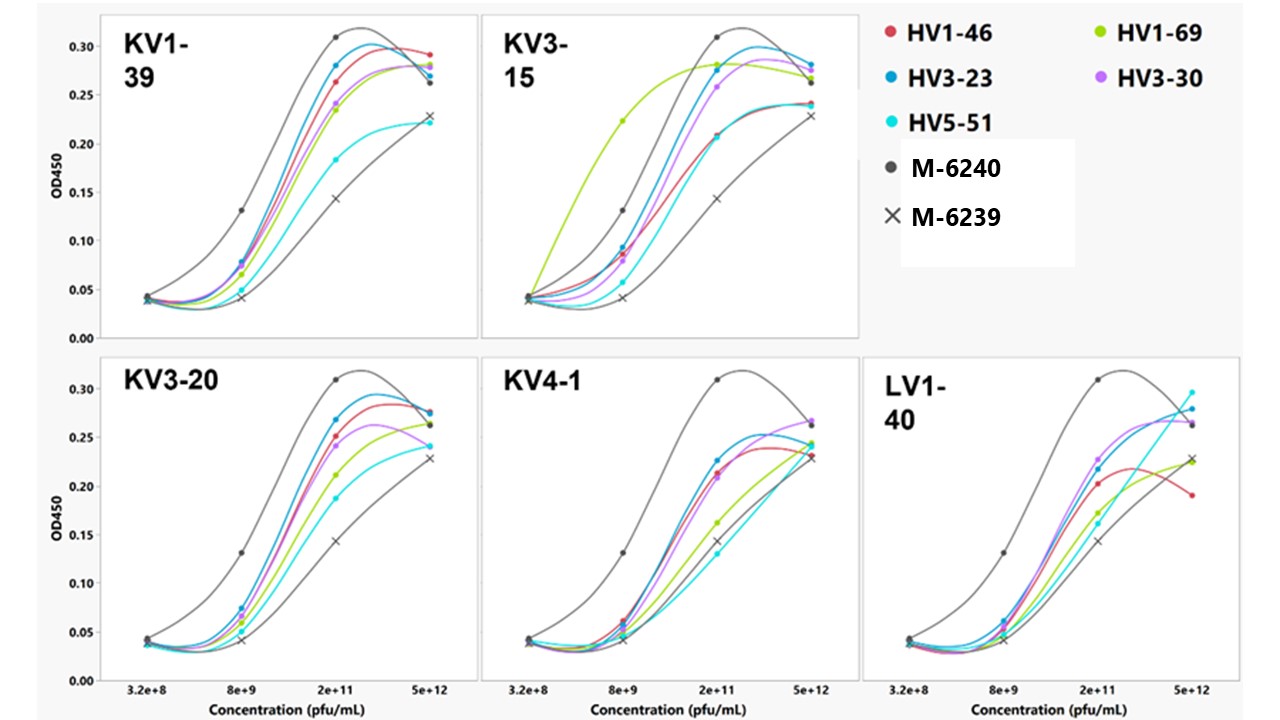


**Supplemental Figure 1. Fab phage display levels of 25 GAN sub-libraries.** ELISA showing Fab expression of 25 GAN sub-libraries from polyclonal phage. Graphs are grouped by the LC germline family. High- and low-expressing Fab on phage controls (M-6240 and M-6239, respectively) are included on each graph as a reference.


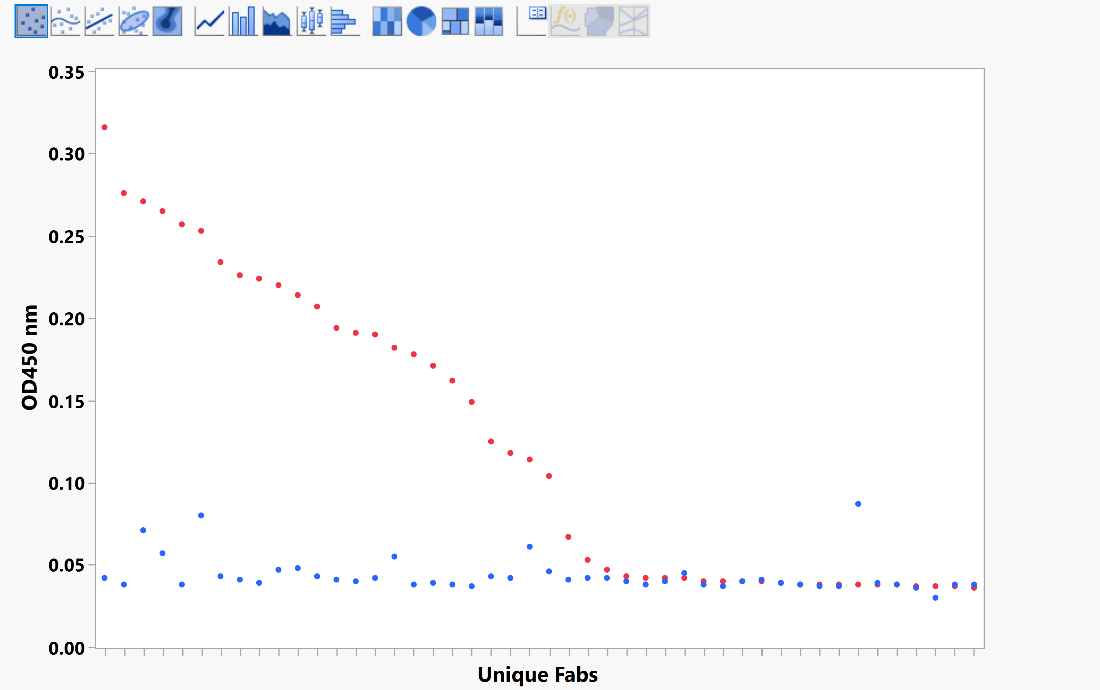


**Supplemental Figure 2. Isolated Fabs display specific binding to SARS-CoV-2 target antigen.** Monoclonal phage ELISA analysis of unique Fab clones enriched after three rounds of selection against RBD. Binding to immobilized RBD (red) or the irrelevant antigen CD40 (blue) are shown. RBD specificity was defined as having RBD binding intensities at least twice that against CD40.


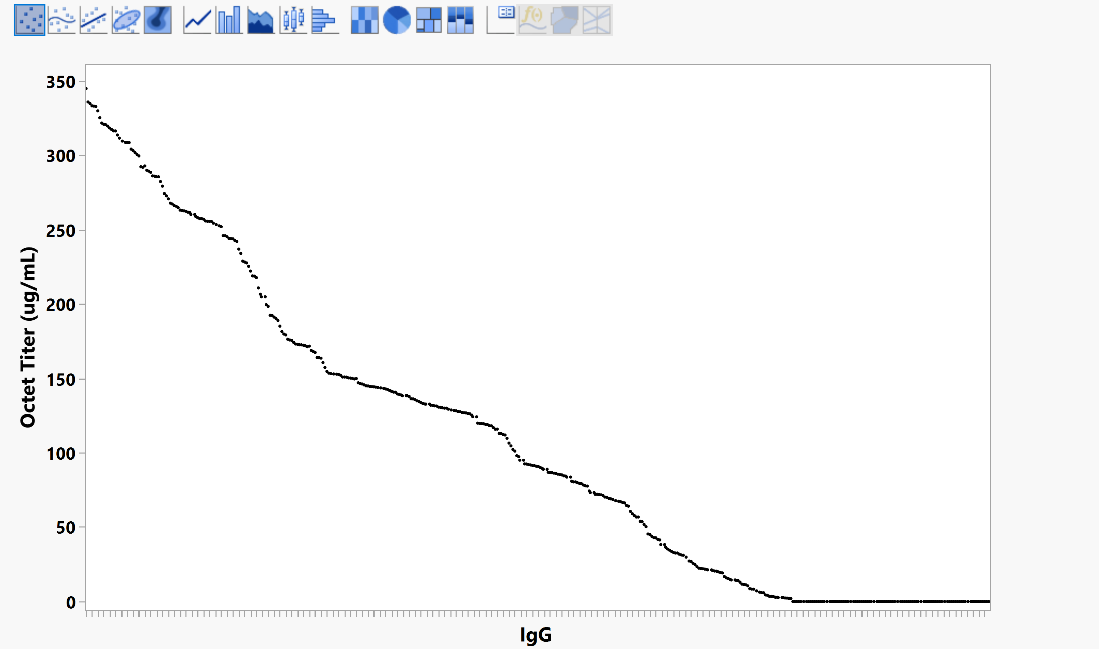


**Supplemental Figure 3.** Expi293F titers of 463 IgG sequences after 4 days of expression, as measured by bio-layer interferometry using the Octet® RED96.


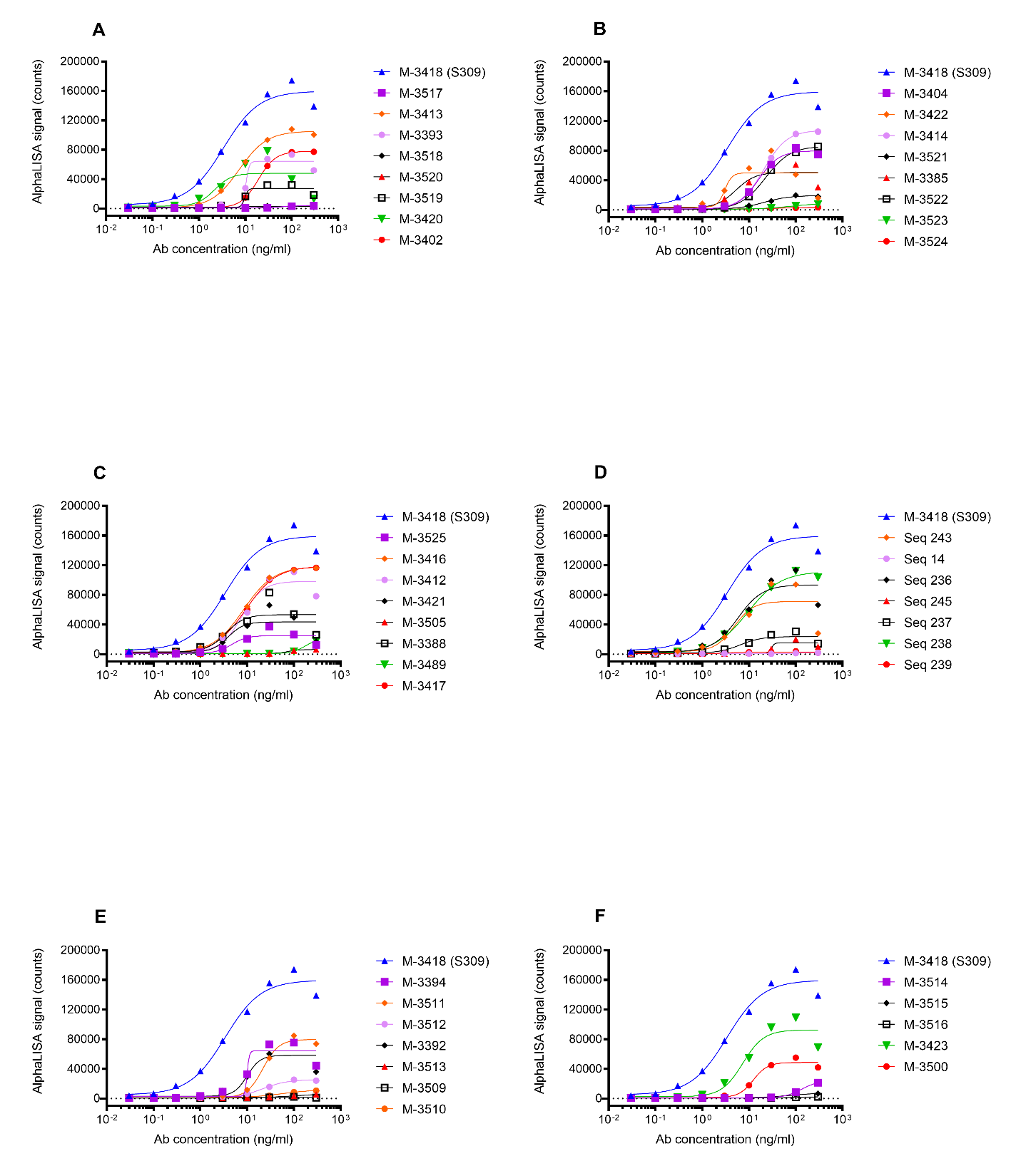


**Supplemental Figure 4. J.HAL**^®^ **IgG Antibodies exhibit dose-dependent binding to SARS-CoV-2 spike protein.** Unpurified transfection supernatants were concentration normalized and tested for binding in a 9-point serial titration series to biotinylated SARS-CoV-2 Spike protein by AlphaLISA. Data was graphed using GraphPad Prism software. Representative binding profiles for 43 J.HAL^®^ antibodies are shown in the sub-plots A-F above, along with the positive control antibody M-3418 (S309).


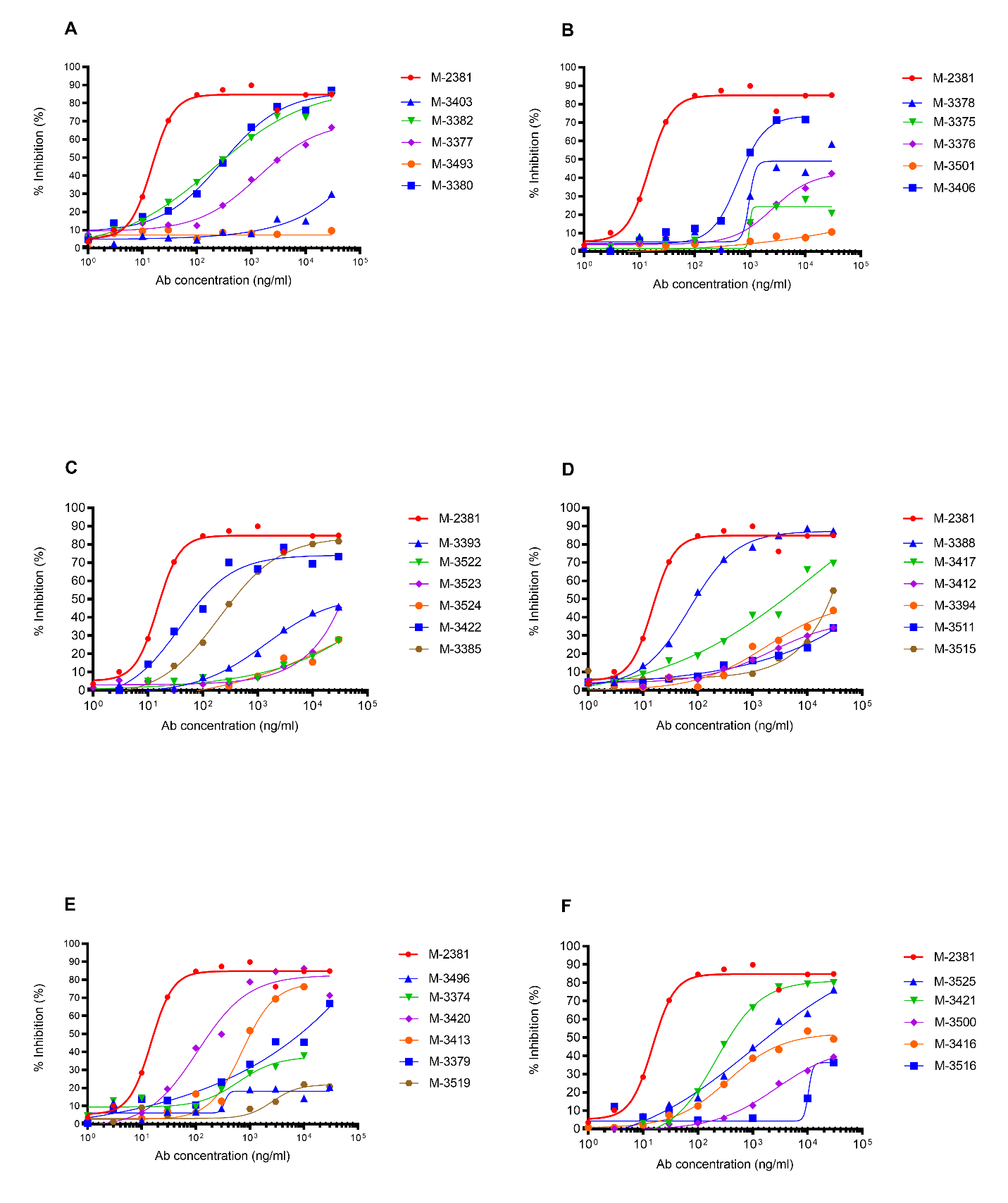


**Supplemental Figure 5. J.HAL**^®^ **IgG Antibodies exhibit dose-dependent blockade of SARS-CoV-2 Spike: human ACE-2 receptor interaction.** Unpurified transfection supernatants were concentration normalized and tested for blocking activity in a 10-point serial titration series. Data was graphed as % Inhibition +/- SD using GraphPad Prism software. Representative binding profiles for 33 J.HAL^®^ antibodies are shown in the sub-plots A-F above, along with the positive control antibody M-2381 are shown.


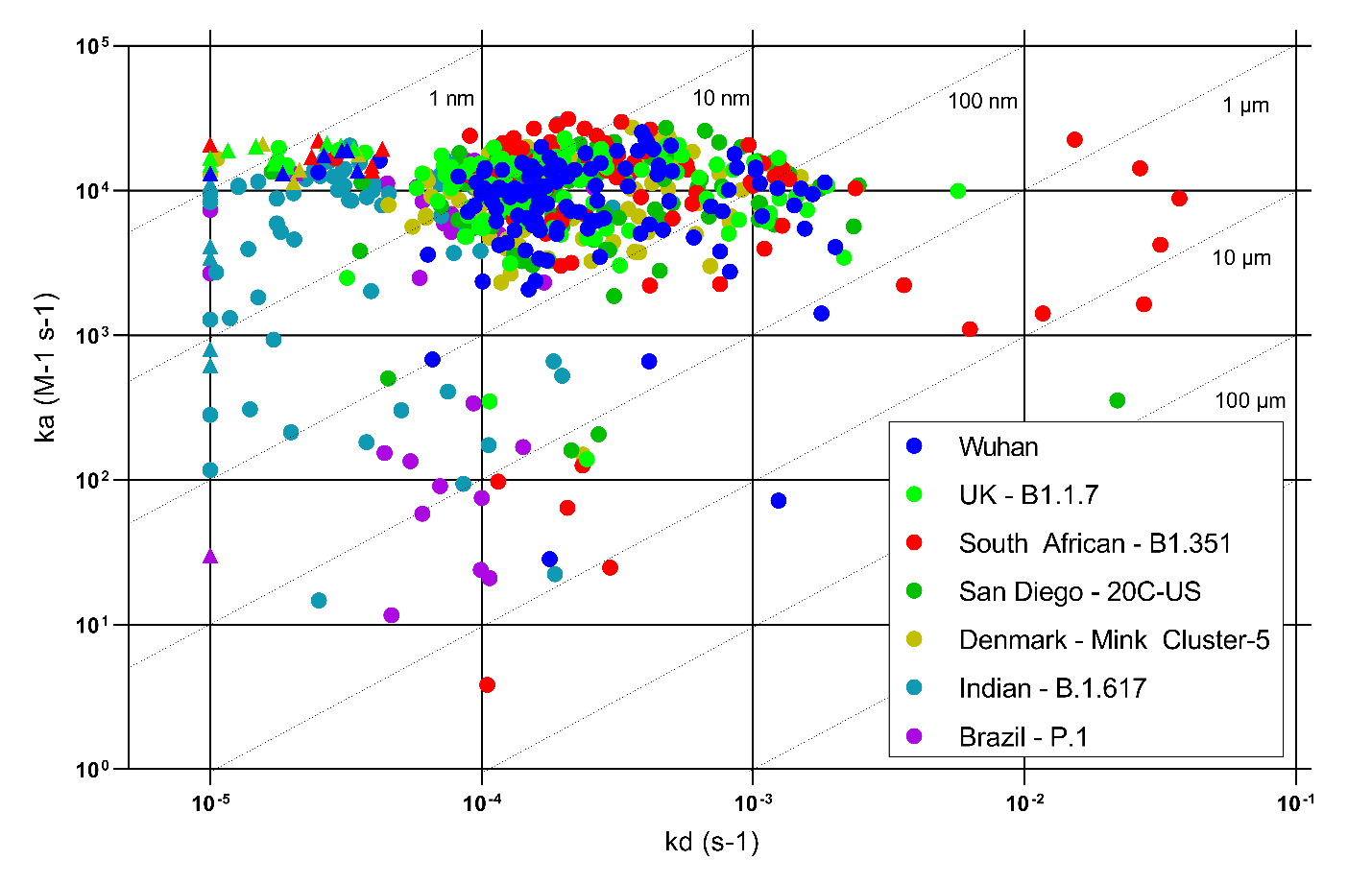


**Supplemental Figure 6. SPR demonstrating cross-reactivity of top J.HAL**^®^ **antibody candidates.** Triangle markers are the S309 control antibody. All other markers on the iso-affinity plot represent one J.HAL^®^ IgG binding interaction with one of the seven SARS-CoV-2 strains as indicated in the legend.

**
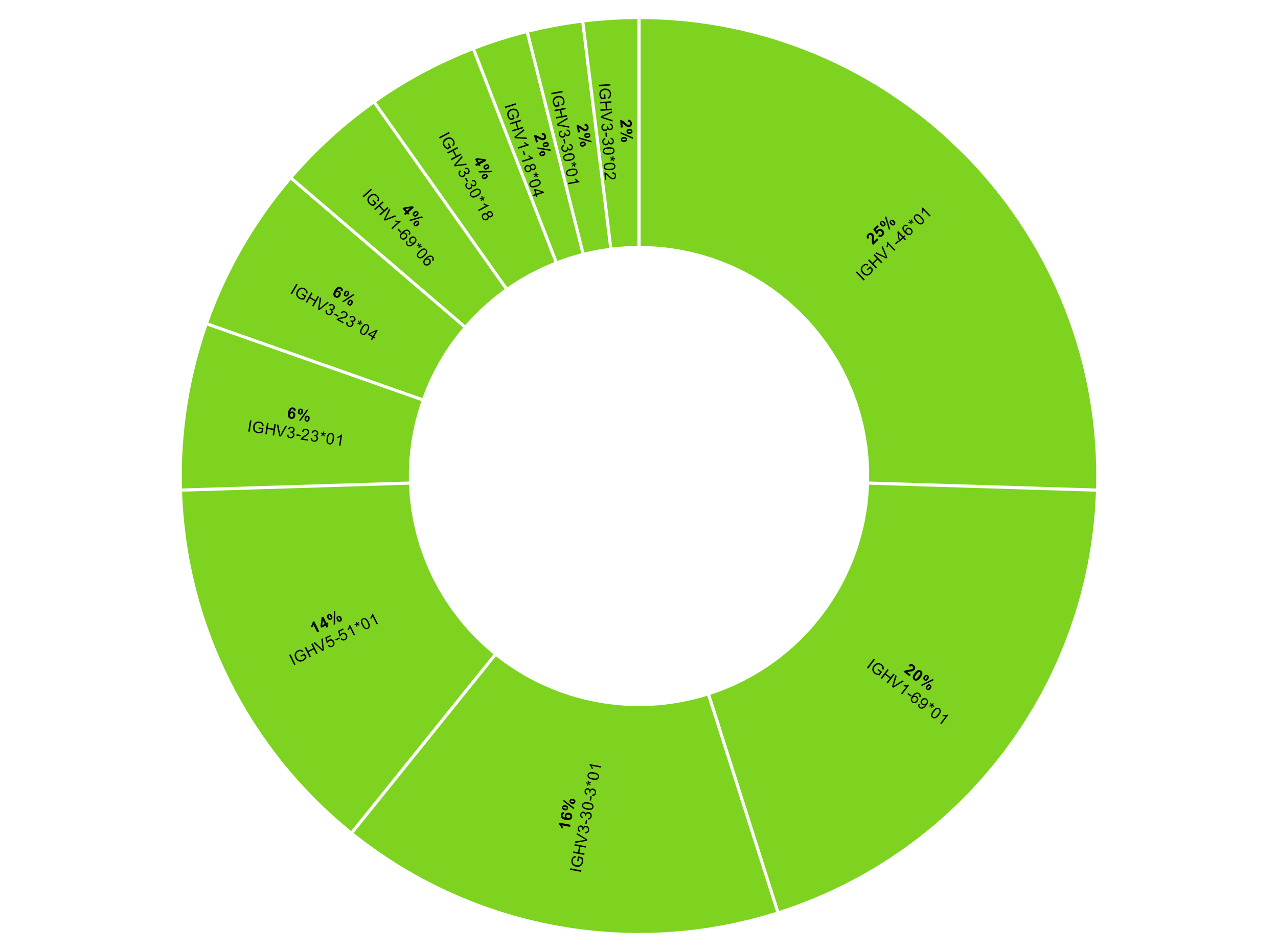
A.**

**B.**


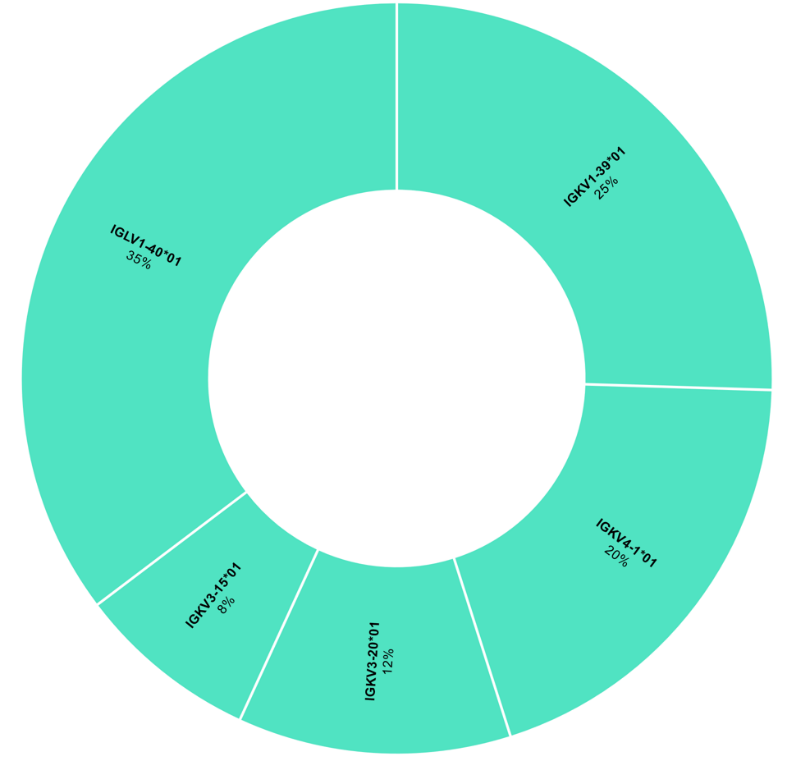


**Supplemental Figure 7. Germline distribution of candidates isolated from J.HAL**^®^ **exhibiting binding specificity to SARS-CoV-2 spike protein.** Diagram in green (A) lists the heavy germline subgroups whereas the diagram in aqua (B) lists the light germline subgroups. Every germline group in the library was represented in the panel of specific binders.

| **Ab** | **EC_50_ (ng/ml)** | **Emax value** |
| --- | --- | --- |
| M-3418 | 4.2 | 121545 |
| M-3395 | 17.0 | 67708 |
| M-3490 | 111.7 | 10407 |
| M-3374 | 26.5 | 43875 |
| M-3375 | 5.8 | 86820 |
| M-3403 | 5.7 | 78177 |
| M-3389 | 4.4 | 53712 |
| M-3397 | 7.3 | 68554 |
| M-3390 | 6.3 | 54307 |
| M-3376 | 8.9 | 75566 |
| M-3396 | 6.3 | 68423 |
| M-3407 | 8.3 | 85746 |
| M-3377 | 12.5 | 56439 |
| M-3406 | 7.2 | 80909 |
| M-3493 | 208.3 | 17156 |
| M-3494 | 42.2 | 6682 |
| M-3378 | n/a | 36011 |
| M-3387 | 6.1 | 52083 |
| M-3379 | 13.3 | 73976 |
| M-3380 | n/a | 38133 |
| M-3496 | 3.2 | 28382 |
| M-3386 | 12.6 | 51958 |
| M-3381 | 22.4 | 49261 |
| M-3498 | 36.0 | 61444 |
| M-3499 | 27.2 | 33902 |
| M-3501 | 14.4 | 4319 |
| M-3502 | 23.1 | 3292 |
| M-3382 | 4.4 | 80143 |
| M-3398 | 10.6 | 69524 |
| M-3503 | 52.6 | 24187 |
| M-3504 | 18.5 | 49166 |

**Supplemental Table 1.** EC_50_ values and Emax values of J.HAL^®^ IgG Antibodies dose-dependent binding to SARS-CoV-2 spike protein.

| **Ab** | **IC_50_ (ng/ml)** |
| --- | --- |
| M-2562 | 15 |
| M-3374 | 613 |
| M-3375 | ~ 978.8 |
| M-3403 | N.C. |
| M-3376 | 2574 |
| M-3377 | 1364 |
| M-3406 | 618 |
| M-3493 | N.C. |
| M-3378 | 964 |
| M-3379 | N.C. |
| M-3380 | 293 |
| M-3496 | ~ 342.1 |
| M-3500 | 2732 |
| M-3501 | N.C. |
| M-3382 | 200 |
| M-3412 | 2215 |
| M-3394 | 1892 |
| M-3511 | N.C. |
| M-3515 | N.C. |
| M-3516 | N.C. |
| M-3519 | 2608 |
| M-3420 | 109 |
| M-3413 | 721 |
| M-3393 | 1408 |
| M-3522 | N.C. |
| M-3523 | N.C. |
| M-3524 | N.C. |
| M-3422 | 38 |
| M-3385 | 203 |
| M-3525 | 997 |
| M-3421 | 217 |
| M-3388 | 70 |
| M-3417 | N.C. |
| M-3416 | 353 |

N.C. = not calculated, due to poor sigmoidal curve fit.

**Supplemental Table 2.** IC_50_ values of J.HAL^®^ IgG Antibodies dose-dependent blocking to SARS-CoV-2 spike protein binding to human ACE-2 receptor protein.
